# Au anchoring on 3D hierarchically porous Ti_3_C_2_T_*x*_ MXene aerogels for enhanced selectivity, response and kinetics in room-temperature trimethylamine sensing

**DOI:** 10.1039/d6ra03153f

**Published:** 2026-05-26

**Authors:** Yongchuan Quan, Ran Zhou, Zhengao Cheng, Xiaoning Qiu, Ling Jin

**Affiliations:** a School of Chemistry and Chemical Engineering, Anhui University of Technology Ma'anshan Anhui 243032 China qxn@ahut.edu.cn jinling@ahut.edu.cn

## Abstract

This study fabricated a 3D Au-anchored Ti_3_C_2_T_*x*_ MXene/sodium carboxymethylcellulose aerogel with a hierarchical porous structure through a facile freeze-drying-assisted strategy. *In situ* redox-driven surface functionalization enables site-specific, uniform anchoring of Au NPs on MXene. The optimized AMC aerogel exhibits a high selectivity of 3.88 and an ultrahigh sensing response of 90.7% at 100 ppm of TMA at room temperature, outperforming most reported MXene-based TMA sensors. It also exhibits excellent long-term stability (10.4% response attenuation over 60 days) and drastically shortened response/recovery time (from 387/682 s to 40.2/162.8 s). Additionally, AMC aerogel is validated for real-time monitoring of seafood (fish and shrimp) freshness by tracking spoilage-related TMA release, providing a promising platform for TMA-based clinical diagnostics. The high TMA sensing performance stems from the synergistic effect between a 3D hierarchical porous structure that provides abundant active adsorption sites and rapid gas diffusion channels, and Au anchoring that modulates the electronic structure of the composite by precisely tuning the d-band center, optimizing Lewis acid-base pairing, and facilitating selective TMA adsorption and fast interfacial charge transfer. This work presents a novel structural design strategy and fundamental insights into high-performance MXene-based aerogels for room-temperature detection of volatile organic compounds.

## Abstract 2

By tuning the structure of MXene, the 3D Au-anchored Ti_3_C_2_T_*x*_ MXene/CMC-Na aerogel sensor detects trimethylamine at room temperature, enabling real-time monitoring of seafood freshness.

## Graphical abstract

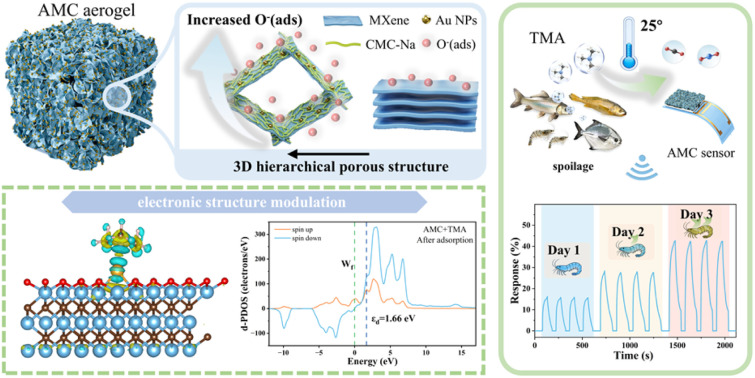

## 1 Introduction

Characteristic volatile organic compounds (VOCs) are emitted during metabolic processes in individuals with diseases and during the microbial spoilage of food.^1,2^ Trimethylamine (TMA), primarily generated from the breakdown of nitrogen-containing organic compounds, is closely linked to impaired liver function in humans and to the spoilage of seafood, including fish and shrimp.^3–5^ Currently, the predominant methods for detecting TMA involve instrumental analyses such as liquid chromatography, spectrophotometry, and mass spectrometry, as well as gas sensors based on metal-oxide semiconductors (MOS).^6,7^ However, traditional instrumental analytical techniques are costly, and the elevated operating temperatures required for MOS sensors limit their applicability in complex room-temperature environments, resulting in diminished response, poor selectivity, and insufficient long-term stability under room-temperature conditions.^8–11^ Electrochemical sensing achieves ultra-sensitive detection of gaseous analytes, providing new insights into the design of high-performance room-temperature gas sensors.^12^

The limited response observed at room temperature is attributed to hindered MOS activation, arising from high surface activation energy at low temperatures, which reduces adsorption affinity and perturbs the adsorbed oxygen species involved in the gain and loss of surface carriers.^13,14^ Additionally, the methyl groups in TMA enhance the electron density around the nitrogen atom through electron-donating induction and hyperconjugation, thereby imparting TMA significant Lewis basicity.^15^ Interfering gas molecules can occupy the active acid-base sites of the sensing materials, disrupting the charge-transfer processes associated with target gases and thereby reducing selectivity.^16–19^ Therefore, achieving high-performance detection of TMA at room temperature remains a significant challenge that warrants thorough investigation.

Transition-metal carbides and nitrides, known as MXenes, exhibit remarkable mechanical stability due to robust covalent bonds between transition-metal and carbon/nitrogen atoms, which give rise to intralayer structures with high crystallinity and minimal defects.^20^ Moreover, the abundance of oxygen-containing groups (–OH/–O) on their surfaces facilitates the formation of strong interlayer hydrogen bonds.^21^ Their distinctive orbital hybridization and 2D layered architecture, enriched with functional terminal oxygen groups, enhance carrier-transport efficiency and interfacial adsorption capacity in MXenes, making them strong candidates for room-temperature chemiresistive sensing.^22^ However, the low selectivity and response of MXene-based gas sensors remain significant challenges that limit their practical applications.^23^

Interface modulation has emerged as a cutting-edge strategy to optimize the room-temperature sensing behaviors of MXene-based composites.^24^ Similarly, defect engineering is proven to strengthen sensing capability, drawing extensive research attention in gas detection.^25^ Constructing heterojunctions by combining metals or semiconductors with MXenes notably enhances charge-transfer and oxygen-adsorption capabilities.^4^ For instance, Quan *et al.*^26^ developed N, C-coordinated Ni–N–C/Ti_3_C_2_T_*x*_ MXene materials using an electrostatic adsorption strategy. The Ni/MXene heterojunction facilitated high carrier transport speed. At the same time, the catalytic activity of Ni and targeted adsorption properties enhanced the NH_3_ sensing signal, resulting in high-performance NH_3_ sensing at room temperature (5 ppm, 27.3%). Additionally, Yang *et al.*^27^ created Fe_2_O_3_/MXene heterojunctions through a straightforward hydrothermal method. The unique layered framework of these heterojunctions enhanced molecular absorption and diffusion, thereby improving response to H_2_S at room temperature. Despite the significant enhancement in the response of MXene-based sensing materials through the construction of heterojunctions, their selectivity for individual molecules remains limited due to the high electron cloud density on the MXene surface and mismatched Lewis acid-base properties.^28–30^

Noble-metal anchoring strategies can significantly enhance selectivity toward molecules with varying Lewis acid-base properties (*e.g.*, strongly basic TMA) by regulating electron-cloud density, tuning the surface d-band center, reconstructing the density of states (DOS) around adsorbed oxygen sites, and modifying interfacial adsorption interactions.^31–33^ Lupan *et al.*^34^ introduced Ag/Pd/AgPt NPs onto the surface of TiO_2_/CuO/Cu_2_O *via* chemical vapor deposition; by adjusting the work function of the material through noble-metal anchoring, they shifted selectivity from ethanol to butanol. Similarly, Li *et al.*^35^ prepared atomically dispersed Pt-anchored Fe_2_O_3_ supports *via* heat treatment under varied atmospheres, and the resulting electronic structure modification increased ethanol selectivity from 1 to 2, affording exceptional sensing selectivity. Kim *et al.*^36^ reported that Au nanoparticles decorated on Lewis acid-base active Cr_2_O_3_ yolk–shell spheres can greatly enhance TMA sensing performance through electronic regulation and catalytic effect, achieving ultrahigh sensitivity. Consequently, combining the high conductivity and mechanical stability of MXenes with the precise electronic regulation afforded by noble-metal anchoring enables meticulous control of electron-cloud density and Lewis acid-base properties at adsorption sites, offering a feasible strategy for realizing molecular selectivity among complex VOCs. Notably, porous mesostructures endow sensing materials with rapid trace detection capability at the ppb level, which is critically important for the precise monitoring of low-concentration harmful gases in practical environments.^37^

In this study, a 3D Au@Ti_3_C_2_T_*x*_ MXene/sodium carboxymethylcellulose (CMC-Na) (AMC) aerogel was synthesized *via* freeze-drying. Au NPs were functionalized and incorporated onto the MXene surface *via* an *in situ* redox reaction, thereby enabling precise control of the surface electronic structure and adsorption sites and enhancing adsorption interactions and carrier transport efficiency. Simultaneously, CMC-Na, a sustainable biopolymer, can interact with MXene *via* hydrogen bonding, facilitating the exfoliation and dispersion of MXene nanosheets and serving as a structural framework for 3D aerogels. The engineered 3D structure leverages the high specific surface area of the 2D materials, resulting in enhanced selectivity and outstanding responsiveness for TMA gas sensing through multifaceted structural regulation. The optimized AMC aerogel exhibited 42% higher TMA selectivity and 247% greater response than unanchored Au NPs for 100 ppm TMA at room temperature. It also maintained stable performance throughout a 60 days stability test, with significantly reduced response and recovery time (from 387/682 s to 40.2/162.8 s). Furthermore, the practical sensing performance of the AMC aerogel was demonstrated in the real-time evaluation of freshness for fresh marine fish and shrimp. Density functional theory (DFT) calculations revealed alterations in the electron cloud structure and energy levels upon noble-metal anchoring, thereby confirming the origin of the enhanced selectivity and response. This study advances gas selectivity and response by meticulously regulating electronic and spatial structures, offering insights and guidance for the development of high-performance VOC-sensing materials.

## 2 Results and discussion

### 2.1 Synthesis and characterization of AMC aerogel

**Scheme 1:**
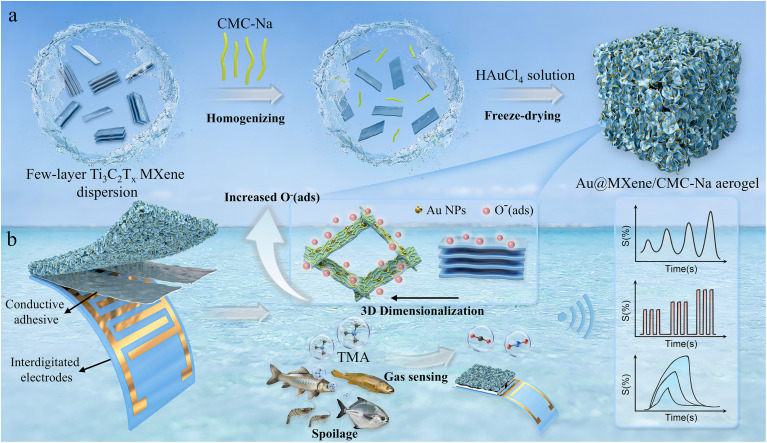
3D AMC aerogels for TMA sensing. (a) Preparation process of 3D hierarchical porous AMC aerogels *via* freeze-drying. (b) The AMC aerogel-based sensor detects TMA in spoiled seafood.

To overcome the inferior sensing performance of pristine MXene-based sensors in detecting TMA at room temperature, a 3D Au-anchored MXene/CMC-Na aerogel with high TMA selectivity and response under ambient conditions was constructed, as shown in Scheme 1. MXene nanosheets tend to self-restack in solution due to interlayer van der Waals interactions. However, the abundant hydroxyl groups on CMC-Na can form strong hydrogen bonds with oxygen-containing functional groups on the MXene surface, enabling uniform dispersion in solution.^38^ Upon contact with Au^3+^ ions, the reductive MXene undergoes an *in situ* redox reaction that induces the growth of Au NPs on its surface. The 3D AMC aerogel is then formed *via* the subsequent freeze-drying process.^39^

The engineered 3D structure effectively maximizes the inherent high specific surface area of 2D MXene materials, significantly increasing the number of available adsorption sites and enhancing the amount of adsorbed oxygen species.^40^ The gas-sensing device was assembled by attaching the aerogel to flexible electrodes with conductive adhesive, enabling real-time detection of TMA released from various seafood samples, which converts chemical signals into visualizable curves and facilitates real-time monitoring of seafood freshness.

**Fig. 1:**
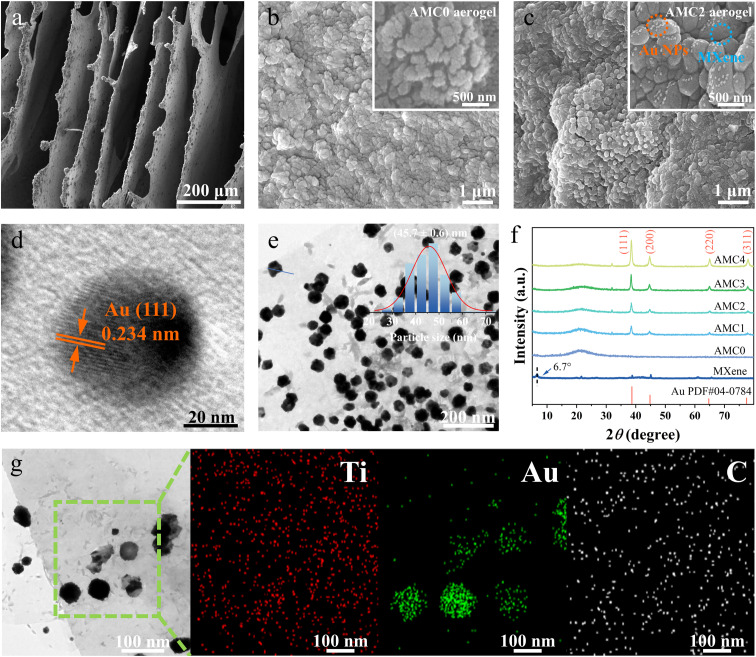
Morphology and composition of AMC aerogels with Au NPs. SEM images of the AMC series aerogels: (a and b) AMC0 aerogel; (c) AMC2 aerogel. (d) High-resolution TEM image of the AMC2 aerogel. (e) Au NP size distribution of the AMC2 aerogel. (f) XRD patterns of Ti_3_C_2_T_*x*_ MXene and AMC0–AMC4 aerogels. (g) TEM-EDS elemental mapping images of Ti, Au, and C in the AMC2 aerogel.

Morphological and structural characterizations of the composite aerogels were performed using SEM, TEM, XRD, and elemental mapping. As illustrated in Fig. 1a and S1, the AMC aerogel exhibits a well-defined layered structure, and after freeze-drying, lamellae with varying stacking orientations interlock to form a 3D hierarchical porous structure. Fig. 1b shows the microstructure of the Au-free AMC0 aerogel, where abundant hydrogen bonds form between MXene lamellae and CMC-Na *via* their surface-enriched oxygen-containing functional groups, facilitating tight stacking of MXene lamellae onto the CMC-Na framework and endowing the composite with a stable 3D conductive network and abundant active sites for gas-molecule adsorption. Fig. 1c shows the microstructure of the optimized AMC2 aerogel, with Au NPs uniformly anchored on the MXene surface, exhibiting excellent dispersibility and no obvious aggregation. When the HAuCl_4_ dosage reached 4 mL, excessive Au NPs aggregated on the MXene surface (Fig. S2). Fig. S3 shows the BET nitrogen adsorption–desorption isotherms of AMC0, AMC1, AMC2, AMC3, and AMC4, while Table S1 presents their corresponding Brunauer–Emmett–Teller (BET) specific surface areas, which are calculated to be 9.01, 12.85, 19.91, 1.23, and 1.00 m^2^ g^−1^, respectively. Pore size distribution profiles of the AMC aerogels are presented in Fig. S4, which shows that the characteristic pore size of AMC2 was approximately 25 Å, smaller than that of AMC0. The findings suggest that the introduction of a low concentration of Au NPs significantly improves the specific surface area of the MXene aerogel. Conversely, excessive Au NP loading induces severe aggregation, which substantially decreases the specific surface area of the aerogel and restricts the exposure and accessibility of active adsorption sites.

High-resolution TEM (HRTEM, Fig. 1d) confirms the crystalline nature of these Au NPs, showing well-defined lattice fringes corresponding to an interplanar spacing of 0.234 nm, consistent with the (111) crystal plane of metallic Au (JCPDS No. 04-0784).^41^ The corresponding SAED pattern (Fig. S5) further verifies the crystalline feature of the prepared material. Statistical particle size analysis (Fig. 1e) yields an average diameter of 45.7 ± 0.6 nm for the Au NPs. These uniformly loaded Au NPs enhance the electron transfer efficiency and specific surface area of the composite, while simultaneously improving gas-molecule adsorption capacity and reaction kinetics at the sensing interface through their intrinsic catalytic and adsorption properties.

XRD patterns of pristine MXene and AMC series aerogels (Fig. 1f) show that the pristine MXene has a characteristic diffraction peak at 6.7° corresponding to its (002) plane.^42^ After CMC-Na incorporation, a broad diffraction peak emerges at ∼22° due to the semicrystalline structure of CMC-Na. In the AMC aerogels, four distinct diffraction peaks at 38.5°, 44.6°, 65.0°, and 77.9° are observed, corresponding to the (111), (200), (220), and (311) crystal planes of Au, respectively.^43^ The intensity of these Au-related peaks increases significantly with rising HAuCl_4_ dosage, indicating increased Au NP loading density. Additionally, elemental mapping of the AMC2 aerogel (Fig. 1g) reveals homogeneous spatial distributions of Ti, Au, and C, further confirming the uniform anchoring of Au NPs on the MXene surface. Overall, these systematic morphological, structural, and compositional characterizations clearly demonstrate the successful fabrication of hierarchically structured AMC aerogels with optimally dispersed Au NPs, which confer favorable structural features and surface properties for high-performance gas sensing.

**Fig. 2:**
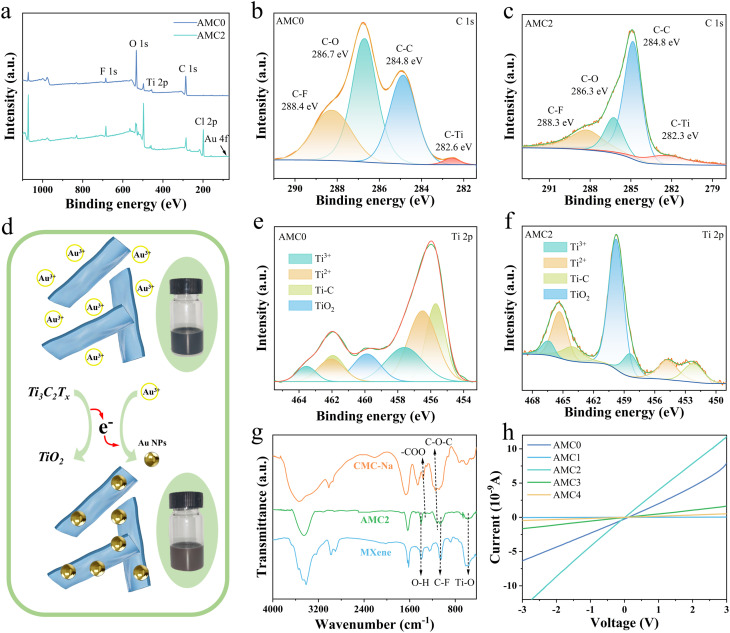
Properties of AMC aerogels. (a) XPS survey spectra of AMC0 and AMC2 aerogels. High-resolution C 1s and Ti 2p XPS spectra are shown for (b and c) AMC0 and (e and f) AMC2 aerogels. (d) Schematic of the *in situ* redox reaction forming Au NPs. (g) FTIR spectra of MXene, CMC-Na, and AMC2 aerogel. (h) *I*–*V* characteristics of AMC0–AMC4 aerogels.

XPS survey spectra were employed to elucidate the surface chemical composition and interfacial bonding states of the samples, with spectra for AMC0 and AMC2 aerogels presented in Fig. 2a. Distinct characteristic peaks corresponding to F, O, Ti, C, Cl, and Au are clearly observed in the spectra. The two peaks in the spectrogram correspond to the 4f_7/2_ and 4f_5/2_ signals of Au, further confirming the loading of Au NPs onto the surface of MXene (Fig. S6). The high-resolution C 1s XPS spectrum of the AMC0 aerogel (Fig. 2b) shows four distinct peaks at binding energies of 288.4, 286.7, 284.8, and 282.4 eV, corresponding to the C–F, C–O, C–C, and C–Ti moieties, respectively.^44^ Following Au incorporation, the high-resolution C 1s spectrum of the AMC2 aerogel (Fig. 2c) reveals a blue shift in the peaks corresponding to the C–O (285.8 eV) and C–Ti (282.2 eV) moieties. Notably, the C l in AMC2 originates from residual HAuCl_4_, and the highly oxidizing Au^3+^ species undergo a redox reaction with the reductive functional groups on MXene nanosheets, which enables the *in situ* growth and uniform immobilization of metallic Au NPs on the MXene surface (Fig. 2d). The high-resolution Ti 2p XPS spectra for AMC0 (Fig. 2e) and AMC2 (Fig. 2f) show characteristic doublets for Ti^2+^, Ti^3+^, and Ti–C species.^45^ With the addition of HAuCl_4_, there is a significant increase in the peak area corresponding to TiO_2_ (459.9 eV), indicating that the low-valent Ti species (Ti^2+^ and Ti^3+^) on the MXene surface are oxidized by Au^3+^ to form TiO_2_ during the redox reaction. Overall, the XPS results confirm the successful *in situ* deposition of Au NPs onto the MXene surface and the formation of strong interfacial interactions between them. These interactions are instrumental in establishing MXene-Au heterojunctions at the sensing interface, which is essential for enhancing gas-sensing performance.

The elemental composition and surface functional groups of the materials were systematically examined *via* Fourier transform infrared (FTIR) spectroscopy (Fig. 2g), allowing identification of the characteristic functional moieties in CMC-Na, MXene, and the AMC2 aerogel. Notably, the characteristic peaks at 1371 cm^−1^ and 1151 cm^−1^ correspond to the –COO– stretching vibrations and the asymmetric C–O–C ether linkage stretching vibrations in CMC-Na, respectively. In addition, the characteristic peaks of MXene observed at 1400, 1057, and 570 cm^−1^ are attributed to the O–H, C–F, and Ti–O stretching vibrations.^46^ All of these characteristic peaks are present in the FTIR spectrum of the AMC2 aerogel, and the redshift of peaks corresponding to –COO– groups and C–O–C ether linkages to lower wavenumbers confirms the formation of hydrogen-bonding interactions between MXene and CMC-Na.

To examine how varying HAuCl_4_ loadings affect the electrical conductivity of the aerogels, current–voltage (*I*–*V*) characteristics measurements were performed on AMC0–AMC4 aerogels, with results shown in Fig. 2h. The electrical conductivity was calculated to be approximately 1.27 × 10^−11^, 5.60 × 10^−14^, 2.35 × 10^−11^, 3.05 × 10^−12^, and 9.22 × 10^−13^ S m^−1^ for the 10 mg mL^−1^ HAuCl_4_ solution with additions of 0, 1, 2, 3, and 4 mL, respectively. The electrical conductivity of the aerogels increases with rising HAuCl_4_ loading at low concentrations but decreases at higher loadings, owing to the transition of Au NPs from uniform dispersion to severe aggregation that disrupts the continuous conductive pathways within the aerogel framework.

To assess the thermal stability of AMC aerogel, Fig. S7 presents the thermogravimetric analysis (TGA) curve of AMC aerogel under nitrogen. Notably, AMC aerogel exhibits a multi-step thermal decomposition process, attributable to the thermal degradation of CMC-Na. Within the temperature range of 300 to 800 °C, the weight loss rate of AMC0 is approximately 44.6%, while those for AMC1, AMC2, AMC3, and AMC4 are 27.4%, 17.8%, 21.3%, and 19.4%, respectively, which is primarily ascribed to interfacial interactions between Au, CMC-Na, and MXene.^47^ The catalytic effect of Au effectively reduces the thermal decomposition rate of the cellulose molecular chains. Additionally, these findings confirm the uniform distribution of Au NPs within the AMC aerogel.

Taken together, the above comprehensive characterizations demonstrate the rational construction of AMC aerogels with strong interfacial binding, homogeneously anchored Au NPs, and favorable electrical properties, providing a fundamental structural basis for the enhanced gas-sensing performance enabled by MXene-Au heterojunctions.

### 2.2 TMA sensing performance of AMC aerogel

**Fig. 3:**
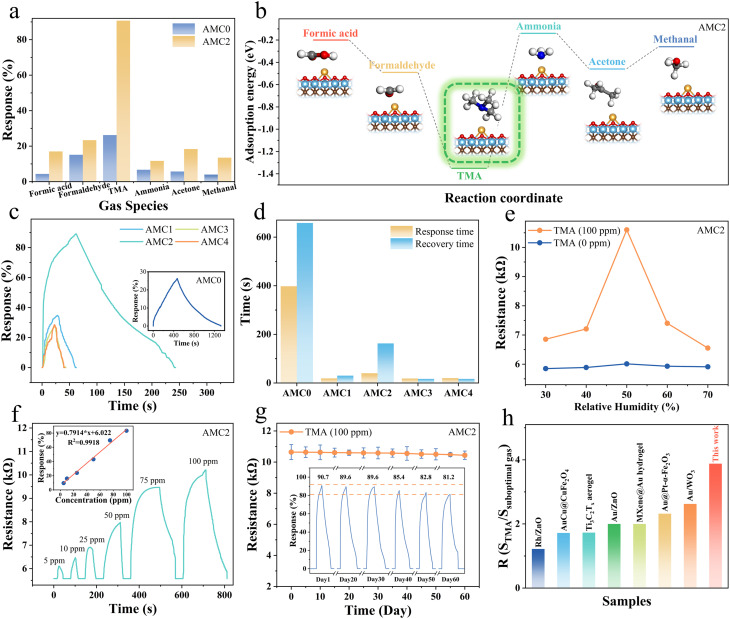
Gas-sensing performance of AMC aerogels. (a) Comparison of selectivity among various sensors. (b) Adsorption energies of the AMC2 sensor for different gases. (c) Response and recovery curves for a range of aerogel-based sensors, with (d) their respective response and recovery time. (e) Resistance of the aerogel under different relative humidity. (f) Dynamic response and recovery curves of the AMC sensor exposed to varying concentrations of TMA, including the correlation fit between TMA concentration and response. (g) Long-term stability of the AMC sensor at 100 ppm TMA. (h) A comparison of sensor selectivity with findings reported in previous literature.

To assess the impact of Au NP anchoring on the sensing performance of the composite material, static testing was employed to systematically investigate and compare the gas-sensing properties of AMC aerogels.^48,49^Fig. 3a shows the selectivity of AMC0 and AMC2 aerogels toward various target gases at room temperature. Selectivity is critical for practical TMA detection, as seafood spoilage environments contain multiple interfering volatile gases. The AMC0 aerogel exhibited the highest response of 26.2% to TMA, while its responses to formic acid, formaldehyde, ammonia, acetone, and methanol were 4.4%, 2.1%, 6.7%, 5.7%, and 4.0%, respectively, indicating excellent TMA selectivity. Following Au incorporation, the AMC2 aerogel exhibited a 90.7% response to TMA, with notably improved responses to other gases as well. Compared with Au-free AMC0 aerogel, the corresponding response values of AMC2 aerogel toward formic acid, formaldehyde, ammonia, acetone, and methanol increased to 22.0%, 33.4%, 16.7%, 23.4% and 18.5%, respectively. The significant enhancement in TMA response upon Au anchoring, coupled with the maintenance of selectivity, prompted further investigation of the underlying mechanism using DFT calculations.

DFT calculations were performed to clarify the intrinsic mechanism of Au NP-modified AMC aerogels excellent TMA selectivity, providing theoretical support for experimental results. Comprehensive DFT calculations were performed to explore the outstanding TMA selectivity of AMC aerogels, through comparison of the adsorption thermodynamics of various responsive gases on the AMC0 surface and the MXene-Au heterojunction in AMC2 (Fig. 3b and S8).^50^ DFT calculations reveal that the TMA adsorption energy of the AMC2 aerogel is −1.35 eV, representing an 110.9% increase compared with that of AMC0 (−0.64 eV, calculated from the absolute value of the adsorption energy). In addition, the adsorption energies of the AMC2 aerogel toward other interferent gases were notably reduced: formic acid (−0.2 eV), formaldehyde (−0.49 eV), ammonia (−0.14 eV), acetone (−0.46 eV), and methanol (−0.26 eV). These adsorption energy differences clearly explain the excellent TMA selectivity of AMC2 aerogels, with the regulatory role of Au NPs further clarified below. The incorporation of Au NPs improved the sensing performance while maintaining the excellent TMA selectivity of the material, and the remarkable difference in binding interaction strength is attributed to the formation of the MXene-Au heterojunction and regulation of the surface electron density by Au NPs.

Subsequently, the response/recovery curves of AMC aerogel sensors toward 100 ppm TMA at room temperature were compared (Fig. 3c). When exposed to TMA, the sensor resistance gradually increased until it reached a stable state, attributed to the n-type semiconductor properties of MXene. AMC0 exhibited a response of only 26.2% to the test gas, with a response/recovery time of 387/682 s, respectively. With increasing HAuCl_4_ solution content, the responses of AMC1, AMC2, AMC3, and AMC4 to TMA increased to 34.9%, 88.9%, 27.9%, and 28.5%, respectively. Among the five as-prepared AMC aerogels with different Au anchoring ratios (AMC0–AMC4), AMC2 delivered the best response and selectivity for TMA and thus exhibited the optimal sensing performance, being employed as the research object for all subsequent experiments. The gas-sensing performance of AMC aerogels mainly depends on the number of MXene-Au heterojunctions formed between MXene and Au NPs. At low Au NPs concentrations, the number of heterojunctions decreases; above a threshold, Au NPs aggregate on the MXene surface, which reduces the contact between TMA molecules and the AMC aerogel interface. The response/recovery time were shortened to 18.8/30.2 s, 40.2/162.8 s, 18.4/16.6 s, and 20.0/16.6 s, respectively, which is attributed to the unique catalytic effect of Au NPs that promotes electron transfer in the reaction (Fig. 3d).^51^ Considering the variable humidity in actual seafood storage environments, investigating humidity stability is necessary to ensure sensor reliability. As shown in Fig. 3e, in the test environment, the sensor response initially increased and then decreased as relative humidity (RH) rose, reaching a peak at 50% RH. When RH is below 50%, water molecules adsorb onto the surface of the sensing material, providing additional adsorption sites and proton-conducting pathways, which enhance sensing performance. However, when RH exceeds 50%, many water molecules aggregate and occupy the original reaction sites, ultimately lowering the response. Unless otherwise noted, all gas-sensing tests in this study were conducted at RH = 50%.

Fig. 3f presents the dynamic response/recovery curves of the AMC aerogel sensor toward different TMA concentrations at room temperature. The sensor exhibited a distinct concentration-dependent response, with excellent detection capability at low TMA concentrations. The fitting results of the sensor response to different TMA concentrations are also shown. For 5, 10, 25, 50, 75, and 100 ppm TMA, the responses were 9.6%, 16.0%, 24.0%, 43.1%, 69.8%, and 90.7%, respectively. Notably, 5 ppm was confirmed as the experimental limit of detection (LOD) of the AMC aerogel sensor, which was the minimum identifiable TMA concentration that could be effectively detected in this measurement.

A linear relationship was observed between the response of the AMC aerogel sensor and TMA concentration (*y* = 0.7914*x* + 6.022, *R*^2^ = 0.9918), indicating good linearity for quantitative detection and further confirming the superior sensing performance of the AMC aerogel sensor. In addition, a 60 days-term stability test reveals only a marginal ∼10.4% attenuation in the sensing response (Fig. 3g), confirming that functionalization of MXene with the CMC framework effectively reinforces the structural stability of the 3D aerogel.^52^ Mechanical stability is essential for the practical application of the sensor, as it may suffer deformation during transportation or use. To validate the structural stability of the AMC aerogel, controlled compressive stress was applied to induce mechanical deformation (Fig. S9). The experimental results reveal that the AMC aerogel can completely recover its pristine morphology after the removal of compressive deformation. In addition, gas sensing measurements were performed on the AMC aerogel under various compressive strains (0–40%) to evaluate the influence of structural deformation on its sensing capability. Subsequent sensing data demonstrate that the AMC-based sensor maintains robust TMA sensing performance even at a compressive strain of 40% (Fig. S10).

Furthermore, the response ratio of the AMC sensor was compared with previously reported values (Fig. 3h).^53–57^ Table S2 further summarizes the comprehensive comparison of resistive sensing performance between the AMC aerogel and other reported TMA sensors. The AMC sensor exhibits a response ratio of 3.88, which exceeds values reported in previous studies, thereby enabling robust and accurate TMA detection in complex multicomponent gas matrices. Collectively, systematic gas-sensing evaluations and DFT calculations demonstrate that the Au-modified AMC aerogel delivers superior TMA-sensing performance featuring high response, excellent selectivity, fast kinetics, good linearity, and long-term stability, which is mainly enabled by the MXene-Au heterojunction and the catalytic effect of Au NPs. This favorable performance lays a solid foundation for the practical application of the sensor in seafood freshness monitoring.

### 2.3 Spoilage testing of seafood conducted by AMC aerogel

**Fig. 4:**
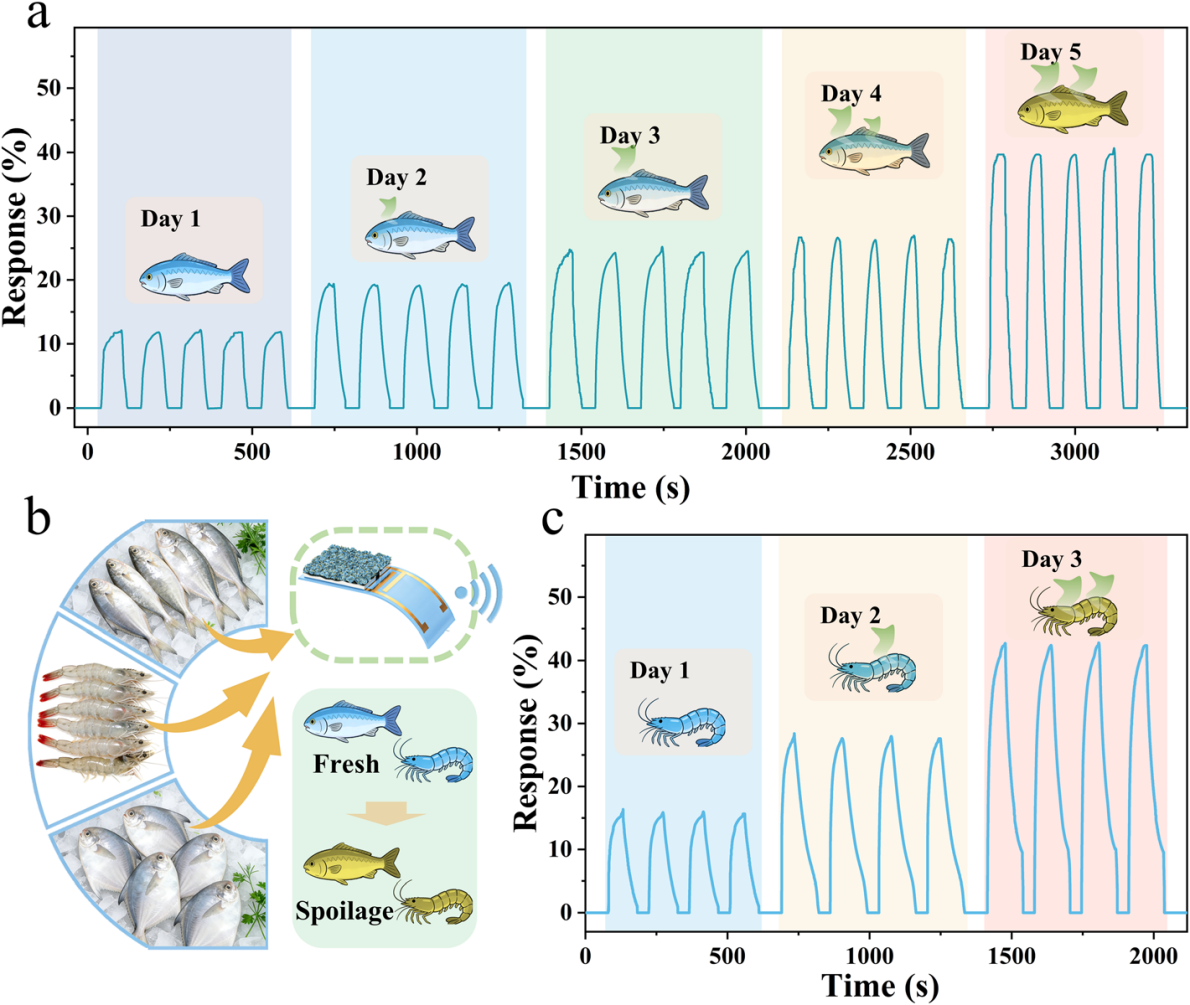
Evaluation of AMC2 sensor for seafood freshness monitoring. (a) Cyclic tests of the AMC sensor for gases released by 100 g of marine fish over 5 consecutive days. (b) Schematic diagram of the AMC sensor for real-time monitoring of seafood freshness. (c) Cyclic tests of the AMC sensor for gases released by 100 g of shrimp for 5 consecutive days.

During seafood spoilage, TMA is released as nitrogen-containing compounds decompose, and changes in its concentration serve as a key indicator of freshness.^5^ Accurately assessing seafood freshness is essential for ensuring food safety. To evaluate the practical applicability of the AMC sensor for real-time monitoring of seafood freshness, 100 g of marine fish were rinsed with deionized water and placed in a 5000 mL test chamber. Cyclic response tests were conducted on the gas within the test chamber over five consecutive days (Fig. 4a). As spoilage progressed, the sensor response increased from 12.5% on day 1 to 40% on day 5, revealing a strong correlation between the spoilage level in marine fish and the sensor response. The excellent cyclic stability of the sensors further indicates that the AMC sensor enables real-time monitoring of seafood freshness (Fig. 4b). Additionally, cyclic tests were carried out on gases released by 100 g of shrimp over three consecutive days (Fig. 4c). Here, the sensor response rose from 16% on day 1 to 42.4% on day 3. The higher TMA levels in shrimp are due to the reduction of TMA N-oxide to TMA during spoilage. Overall, these results confirm the potential of the AMC sensor for real-time monitoring of seafood freshness.

### 2.4 Gas sensing mechanism

**Fig. 5:**
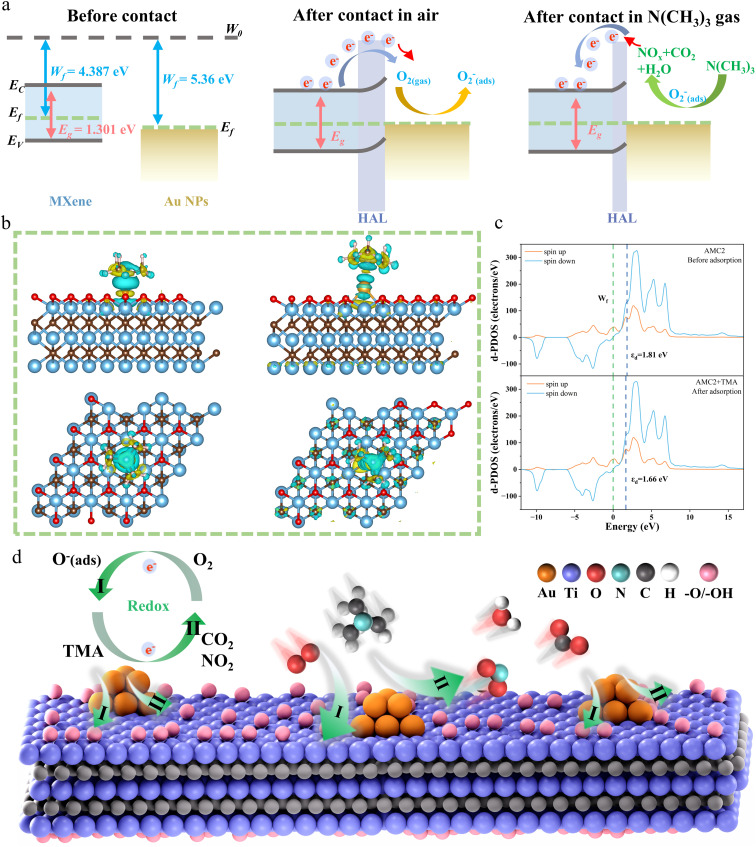
Theoretical analysis of electronic structure and TMA sensing mechanism for AMC aerogel. (a) *W*_f_ and *E*_g_ of MXene and Au, as well as the band structures of AMC aerogel in air and TMA. (b) Charge-density differences between the MXene and MXene-Au after TMA adsorption. (c) DOS and d-band centers (including spin-up and spin-down) of AMC aerogel before and after TMA adsorption. (d) Schematic diagram of the sensing mechanism of AMC aerogel toward TMA.

The sensing mechanism of the AMC aerogel is attributed to a classic, widely accepted heterostructure, in which a hole-accumulation layer (HAL) forms *via* electron transfer, and the target gas undergoes a redox reaction with surface-adsorbed oxygen species on the sensing material.^58^ Specifically, the high selectivity and response of the AMC aerogel are attributed to the regulation of the electronic structure induced by Au anchoring and the increased amount of adsorbed oxygen species derived from the 3D hierarchical porous structure. The proposed gas sensing mechanism is illustrated in Fig. 5a. As a typical n-type semiconductor (Work function (*W*_f_) = 4.7 eV, Band gap (*E*_g_) = 1.301 eV), MXene forms a heterojunction upon contact with Au NPs (*W*_f_ = 5.36 eV).^59^ To achieve thermodynamic equilibrium, electrons transfer from MXene with a higher Fermi level to Au NPs with a lower Fermi level, and a HAL is formed on the MXene surface due to trapped electrons.^60^

When the sensor is in air, oxygen is adsorbed on the surface of the sensing material, and these adsorbed oxygen molecules acquire electrons through the following processes to generate oxygen anion species:

1 O_2_(gas) → O_2_(ads)

2 O_2_(ads) + e^−^ → 2O^−^/O_2_^−^(ads)

These adsorbed oxygen species capture electrons from MXene, thereby decreasing the sensor resistance in air. When the sensing material is exposed to basic TMA gas, oxygen anions on its surface undergo redox reactions with TMA molecules, releasing the trapped electrons back to the HAL. Consequently, the released electrons recombine with holes in the HAL, which reduces the HAL thickness and further increases the resistance of the sensing material.^61^ The reaction process is as follows:

3 2N(CH_3_)_3_ + 23O^−^ → 2NO_2_ + 6CO_2_ + 9H_2_O + 23e

To further elucidate the excellent selectivity of the AMC aerogel toward TMA, the charge density difference (CCD) of MXene and MXene-Au upon TMA adsorption was systematically explored and analyzed. As shown in eqn (4), the CCD is defined as the value obtained by subtracting the charge density of the gas molecule before adsorption and the charge density of the MXene (Au) surface before adsorption from the charge density of the system after TMA adsorption:

4 CCD = *C*_(TMA+MXene−Au)_ − *C*_(TMA)_ − *C*_(MXene−Au)_

The CCD results are shown in Fig. 5b, where yellow electron clouds represent charge accumulation regions and blue electron clouds indicate charge depletion regions.^50^ CCD analysis reveals a large charge depletion region between MXene and TMA, accompanied by significant charge transfer from the MXene-Au heterojunction to TMA, indicating a strong chemical interaction between the MXene-Au heterojunction and TMA.^50,62^ Collectively, the Au-anchoring-induced modification of the charge structure helps regulate the Lewis acid-base properties on the MXene-Au surface, thereby improving its selectivity for TMA (a gas with strong Lewis basicity) and endowing the AMC aerogel with excellent TMA selectivity.

To further rationalize the high response of the AMC aerogel toward TMA, the electronic structure and the concentration of adsorbed oxygen species in MXene-Au were analyzed. The site-dependent total density of states (DOS) of the AMC aerogel was analyzed to understand its electronic structure (Fig. 5c). In the AMC model, the d-band center shifts downward from 1.81 to 1.66 eV after TMA adsorption. As an electron donor, TMA preferentially fills the Au 5d orbitals (which have a high work function) while modulating the local electron density at the MXene-Au interface, thereby shifting the d-orbital energy level of the entire system to lower energy.^63–65^ Consequently, the synergistic interaction between Au and MXene effectively accelerates the charge-transfer kinetics during the redox process. Additionally, Au anchoring modulates the activation energy (*E*_a_) of the adsorption reaction by altering the charge transfer behavior between the adsorbates and the material surface. The activation energy is calculated using the following Arrhenius equation (eqn (5)):

5 *S* = exp(−*E*_a_/*kT*)

where *S* denotes the response (*R*_g_/*R*_a_), *E*_a_ is the activation energy, *k* is the Boltzmann constant, and *T* represents the operating temperature. The calculated results demonstrate that the activation energy of the optimized AMC aerogel is significantly lower than that of the pristine sample (Fig. S11). The reduction in activation energy improves the efficiency of the adsorption process, thereby increasing the response and decreasing the response/recovery time.

To examine the impact of adsorbed oxygen (O_c_) on the gas-sensing performance of the aerogels, the O 1s XPS spectra of pristine MXene, blank sample, and AMC aerogel are shown in Fig. S12. The spectra show two fitted peaks corresponding to O_c_ and lattice oxygen (O_L_), respectively.^66^ The relative O_c_ content in pristine MXene is 41.2%, significantly lower than that of AMC0 (64.6%) and AMC2 (68.1%). These findings indicate that constructing a 3D architecture from 2D MXene nanosheets effectively increases the number of active adsorption sites for oxygen molecules.^48^ Additionally, incorporating an appropriate amount of Au NPs further increases the density of these sites.

In summary, the excellent TMA-sensing performance of the AMC aerogel is attributed to Au-induced electronic modulation and the unique 3D hierarchical porous structure, both of which jointly enhance electron transfer efficiency, promote the formation of active oxygen species, and reduce the activation energy of surface reactions (Fig. 5d). Verified by both experimental results and DFT calculations, the synergistic effect of Au (regulating Lewis acid-base properties and accelerating electron migration) and MXene (serving as the main electron donor), together with the 3D hierarchical porous structure that provides abundant active adsorption sites, collectively improves the sensor response and selectivity toward TMA.

## 3 Conclusions

The 3D Au-anchored MXene/CMC-Na aerogel was rationally designed and developed into a high-performance room-temperature TMA gas sensor, enabling real-time monitoring of seafood freshness. The resulting AMC sensor exhibits exceptional sensing performance with a high selectivity factor of 3.88 against 1.73 for pristine MXene, a remarkable response of 90.7% toward 100 ppm TMA, excellent long-term stability with merely 10.4% response attenuation over 60 days, and fast response/recovery kinetics of 40.2/162.8 s. Such outstanding TMA-sensing capabilities arise from two synergistic effects: (1) electronic modulation of MXene by Au NPs, which reinforces the selective affinity toward TMA molecules; and (2) the 3D hierarchical porous architecture constructed by the CMC-Na framework, which affords abundant active adsorption sites. DFT calculations further corroborate that charge density redistribution and d-band center downshift induced by Au anchoring are the critical factors responsible for the enhanced sensing performance. This work thus establishes a rational design strategy for high-performance room-temperature TMA sensors, which holds tremendous practical potential for on-site seafood safety supervision and clinical health diagnostics, given the close correlation between abnormal TMA levels and human health disorders.

## 4 Experimental section

### 4.1 Materials

The reagents included Ti_3_C_2_T_*x*_ MXene (10 mg mL^−1^), which was purchased from 11 Technology Co., Ltd (China). CMC-Na (RG) and tetrachloroauric (III) acid hydrate (HAuCl_4_·2H_2_O, 99%) were purchased from Adamas (China). Conductive adhesive (silver paste, purity: 52%) was purchased from Shenzhen Wowis Electronic Technology Co., Ltd (Shenzhen, China). All target gases, including trimethylamine (C_3_H_9_N), formic acid (HCOOH), ammonia (NH_3_), formaldehyde (HCHO), methanol (CH_3_OH), and acetone (C_3_H_6_O), were supplied by Sinopharm Chemical Reagent Co., Ltd.

### 4.2 Synthesis of AMC aerogels

AMC aerogels were synthesized by freeze-drying. First, 0.04 g of CMC-Na was dispersed uniformly in 4 mL of deionized water to yield a homogeneous aqueous solution. Next, 1 mL of a 10 mg mL^−1^ Ti_3_C_2_T_*x*_ MXene dispersion was added, and the mixture was stirred continuously for 30 min. Subsequently, varying volumes (0, 1, 2, 3, and 4 mL) of a 10 mg mL^−1^ HAuCl_4_ aqueous solution were added, and DI water was supplemented to adjust the total volume to 8 mL. After stirring continuously for 1 h, the mixture was degassed under vacuum and transferred to a silica mold. Finally, the mixture was frozen and then freeze-dried to obtain AMC aerogels, denoted AMC0, AMC1, AMC2, AMC3, and AMC4, corresponding to HAuCl_4_ volumes of 0, 1, 2, 3, and 4 mL, respectively.

### 4.3 Characterization

Field-emission scanning electron microscopy (FE-SEM, Zeiss Sigma 500, Germany) and transmission electron microscopy (TEM, JEOL JEM-2100F, Japan) were used to characterize the morphological features and nanoparticle distribution of the samples. X-ray diffraction (XRD, Bruker D8 Advance, Germany) with Cu Kα radiation (*λ* = 1.5406 Å) was used to analyze the crystal structure at a scanning rate of 5° min^−1^. Fourier transform infrared (FTIR) spectroscopy was performed on a PerkinElmer spectrometer (USA) over the wavenumber range of 4000–400 cm^−1^. X-ray photoelectron spectroscopy (XPS, Kratos Axis Supra+, Shimadzu, Japan) was used to investigate the surface elemental composition and chemical bonding states of the aerogels. The specific surface area and pore size distribution of the aerogels were determined by Brunauer–Emmett–Teller (BET) analysis using a Micromeritics ASAP 2460 instrument (USA). Thermogravimetric analysis (TGA, Shimadzu DTG-60H, Japan) was conducted under a nitrogen atmosphere from 30 to 800 °C at a heating rate of 10 °C min^−1^. Current–voltage (*I*–*V*) characteristics were recorded using a CHI 760E electrochemical workstation (Model A18501) to determine the electrochemical properties of the samples.

## Author contributions

Y. Q. conducted the measurements, carried out data analysis, and drafted the initial manuscript. L. J. designed and executed the experiments, performed data interpretation, participated in the discussion of experimental results, and revised the manuscript. R. Z. and Z. C. undertook the computational work of the study. X. Q. contributed to revising the manuscript for clarity and coherence, and assisted in formulating the responses to the reviewers' comments. All authors have reviewed and approved the final version of the manuscript.

## Conflicts of interest

There are no conflicts to declare.

## Supplementary Material

RA-016-D6RA03153F-s001

## Data Availability

The authors confirm that the data supporting the findings of this study are available within the article and its supplementary information (SI). Supplementary information: assembly of gas sensors and preparation procedure of sample gases; *I*–*V* curve measurement and conductivity calculation of the composite material; details of the density functional theory calculation; SEM images of AMC aerogels, SAED of the AMC aerogel, nitrogen adsorption–desorption isotherms of AMC0, AMC1, AMC2, AMC3 and AMC4 aerogels, BJH pore-size distribution plots, TG curves, *E*_a_, high-resolution Au 4f and O 1s XPS spectra; images of the AMC aerogel before and after compression, response curves; adsorption energies of the AMC0 sensor for different gases. See DOI: https://doi.org/10.1039/d6ra03153f.
