# Supplementary material for: Au anchoring on 3D hierarchically porous Ti3C2Tx MXene aerogels for enhanced selectivity, response and kinetics in room-temperature trimethylamine sensing
Source: RSC Adv. 2026 May 26;16(31):28423–34. doi: 10.1039/d6ra03153f (PMC13213552; doi:10.1039/d6ra03153f)
Supplement: RA-016-D6RA03153F-s001 [file RA-016-D6RA03153F-s001.pdf]

Supplementary Information (SI) for RSC Advances.
This journal is © The Royal Society of Chemistry 2026

Supporting Information

Au Anchoring on 3D Hierarchically Porous $Ti_3C_2T_x$ MXene Aerogels for Enhanced Selectivity, Response and Kinetics in Room-Temperature Trimethylamine Sensing

Yongchuan Quan [1], Ran Zhou [1], Zhengao Cheng [1], Xiaoning Qiu[1]* and Ling Jin [1]*

[1] School of Chemistry and Chemical Engineering, Anhui University of Technology, Ma'anshan, Anhui 243032, China

Correspondence: qxn@ahut.edu.cn (X.N. Qiu), jinling@ahut.edu.cn (L. Jin)

Assembly of Gas Sensors and Preparation procedure of Sample Gases

AMC aerogel sensors were fabricated by uniformly applying 0.2 mm-thick conductive silver paste to both ends of the as-prepared aerogels to prevent short circuits. The treated aerogels were then attached onto flexible interdigital electrodes (25 electrodes; length: 10 mm, width: 0.35 mm, spacing: 50 μm) supported by a 13 μm-thick PI substrate. The assembly was dried at 60 °C for 3 h to fully cure the conductive silver paste, affording the complete gas sensor element. Sensing performance was characterized using the static liquid-gas distribution method. The liquid volume corresponding to the target gas concentration was calculated according to Equation S1:

$$C_m = \frac{22.4 \times d \times \rho \times V_l^{'}}{V_b \times M_m \times 200} \times \frac{273.15 + T}{273.15} \times 10^9 \quad \text{(S1)}$$

The designed gas concentration within the test chamber C_m (ppm), liquid density d ($g \cdot cm^{-3}$), required liquid solution volume V_l' (mL), liquid reagent purity ρ (mass fraction), molar mass of the target gas M_m ($g \cdot mol^{-1}$), test chamber volume V_b (mL), and gas temperature T (°C) are defined as above. The gas was allowed to cool naturally to room temperature (15 °C, winter conditions). The sensor was first placed in ambient air until its resistance stabilized at R_a. Test gas was then introduced, and the steady-state resistance R_g was recorded after equilibration. This process was repeated, and the sensor response was defined as $S=|R_a-R_g|/R_a$. Response and recovery time were defined as the time required for the sensor resistance to reach 90% of its total variation upon gas exposure and removal, respectively. Selective definition refers to the TMA/interferent response ratio.

I–V curve measurement and conductivity calculation of the composite material

Current–voltage (I–V) characteristics were acquired using an electrochemical workstation (Model CH1760E A18501) at room temperature. The AMC aerogel supported on interdigital electrodes was scanned from −3 to 3 V at a rate of 0.05 $V \cdot s^{-1}$. A bias voltage of 0 V was maintained at the onset and conclusion of measurements to avoid irreversible polarization. The interdigital electrode consisted of 25 pairs of ITO electrodes on a 13 μm-thick PI substrate (10 × 10 mm^2), with electrode width and interelectrode gap both fixed at 50 μm and an overlapping length of 30 mm. The

electrical conductivity (σ) of the composite was calculated using Equation S2 (given below)[1]:

$$\sigma = \frac{\mathrm{d} \times I}{(2n-1) \times L \times h \times v} \tag{S2}$$

where d denotes the interelectrode spacing, I and V correspond to the current and voltage, respectively, n stands for the number of electrode fingers, and L refers to the overlapping length of the electrodes. If the aerogel thickness exceeded that of the ITO electrodes, L was taken as the electrode thickness; otherwise, h represented the aerogel thickness.

Details of the Density functional theory (DFT) calculation

Computational Models Construction: This study investigates the effects of Au loading on the structural and electronic properties of two-dimensional MXene materials via a supercell model of Au-modified $Ti_3C_2T_x$. A $Ti_3C_2T_x$ MXene substrate model was first constructed with lattice constants of 12.266 Å (X and Y directions) and 19.720 Å (Z direction), incorporating Ti, C, and surface-active O atoms. Au atoms were subsequently introduced to form an MXene-Au composite model, to simulate Au adsorption on the MXene surface and examine charge transfer behaviors. All models were optimized using DFT to obtain equilibrium configurations. The results demonstrate that Au atoms can stably adsorb on the MXene surface while preserving structural integrity, providing a computational foundation for future applications in catalysis and sensing. This modeling strategy is effective: the 12.27 Å supercell size avoids spurious interactions between Au atoms, and the 19.720 Å Z-axis dimension ensures a sufficient vacuum layer to eliminate interlayer interference. Moreover, DFT enables accurate descriptions of electronic orbital hybridization and bond-length variations between Au and $Ti_3C_2T_x$ surface atoms, guaranteeing the validity of the calculated results.

Computational Parameters and Methods: All first-principles calculations were conducted via the CASTEP package within the framework of DFT. Geometric structure optimization was carried out using the Perdew–Burke–Ernzerhof (PBE) exchange–correlation functional under the generalized gradient approximation (GGA), which

offers a balance between accuracy and efficiency for describing metal oxide adsorption and electronic properties. To address local electronic correlation effects, the DFT+U correction scheme was employed to enhance the localization of transition metal d-orbitals.[2] Additionally, the collinear method was used to account for spin polarization, with the formal spin state designated as the initial spin state to validate the magnetic state.

In the electronic structure calculations, an energy cutoff of 450 eV for the plane-wave basis set was chosen to ensure adequate wave function expansion and reliable convergence of the total energy.[3] Brillouin zone integration used the Monkhorst–Pack method with a 5 × 5 × 4 k-point grid. Convergence tests indicated that this k-point resolution produced a total energy variation of less than 10^{-5} eV per atom, confirming grid sufficiency for structure optimization and electronic property analysis.

To prevent spurious interactions from periodic images along the Z-axis, a vacuum layer of 15 Å was introduced, effectively minimizing interlayer coupling. These settings ensured high accuracy while reducing potential errors arising from finite-size effects, incomplete basis sets, or insufficient Brillouin-zone integration. Norm-conserving pseudopotentials were employed, and relativistic effects were treated using the Koelling–Harmon approximation, thereby balancing computational complexity and accuracy.

Adsorption energy (E_{ads}) was calculated to evaluate material stability for target gas molecule adsorption[4]. The formula is expressed in Equation (S3):

$$E_{ad} = E_{(M-)AMC-gas} - E_{gas} - E_{(M-)AMC} \quad \text{(S3)}$$

In the formula, $E_{(M-)\ AMC-gas}$ represents the total energy of the material in the gas adsorption model, where target gas molecules are adsorbed. E_{gas} represents the total energy of the gas molecules alone, while $E_{(M-)AMC}$ represents the total energy of the material when no gas molecules are adsorbed. All these energies, including $E_{(M-)\ AMC-gas}$, E_{gas}, and $E_{(M-)AMC}$, are expressed in kcal·mol^{-1}. The differences among these energies enable the assessment of adsorption performance, such as the stability of the gas adsorption process and the interaction between the material and the gas molecules.

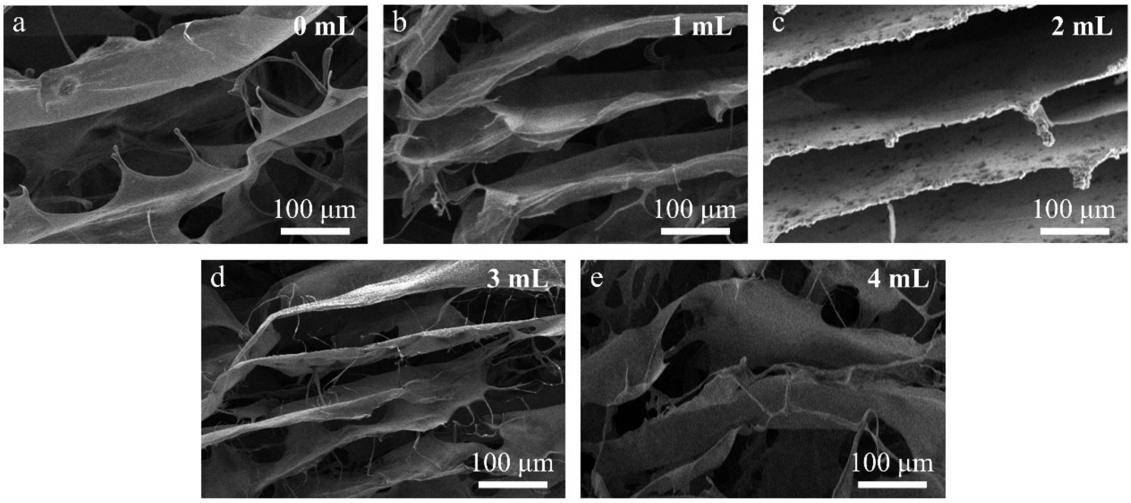

Figure S1. SEM images of AMC aergel with different concentrations of $HAuCl_4$: (a) 0 mL, (b) 1 mL, (c) 2 mL, (d) 3 mL, (e) 4 mL.

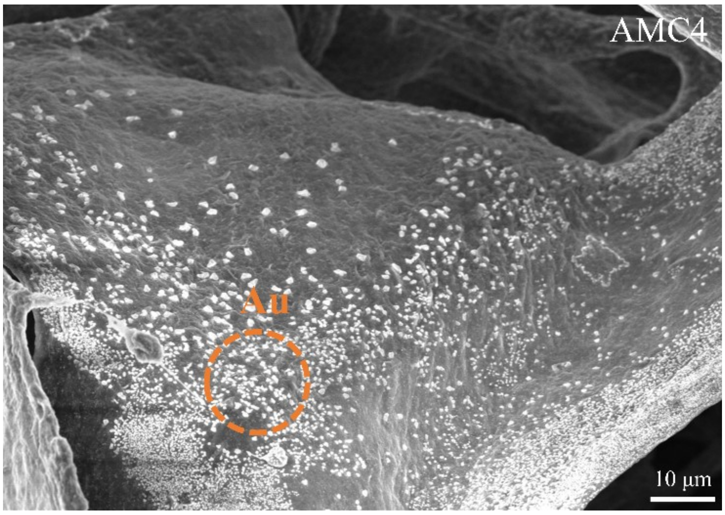

Figure S2. SEM images of AMC4.

Note: Excessive Au NPs aggregate on the surface of MXene at a 4 mL addition of 10 $mg{\cdot}mL^{-1}$ $HAuCl_4$, and the over-agglomerated Au clusters obscure the adsorption sites on the surface of MXene.

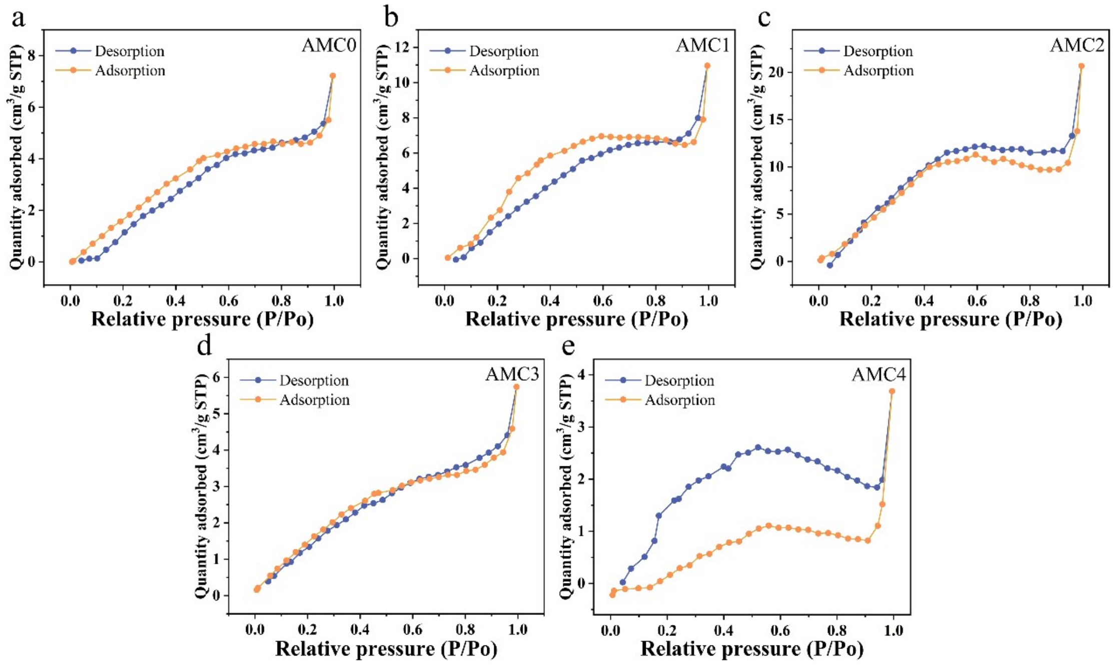

Figure S3. Nitrogen adsorption–desorption isotherms of AMC0, AMC1, AMC2, AMC3 and AMC4 aerogels.

Note: The nitrogen adsorption–desorption isotherms of AMC0–AMC4 aerogels have been shown in Figure S3. All samples exhibit typical Type IV adsorption isotherms, indicating a strong interaction between the material and nitrogen molecules, as well as the presence of mesoporous structure. The calculated BET specific surface areas of AMC0 to AMC4 are 9.01, 12.85, 19.91, 1.23 and 1.00 m^2/g, respectively. The relevant specific surface area parameters have also been summarized in Table S1.

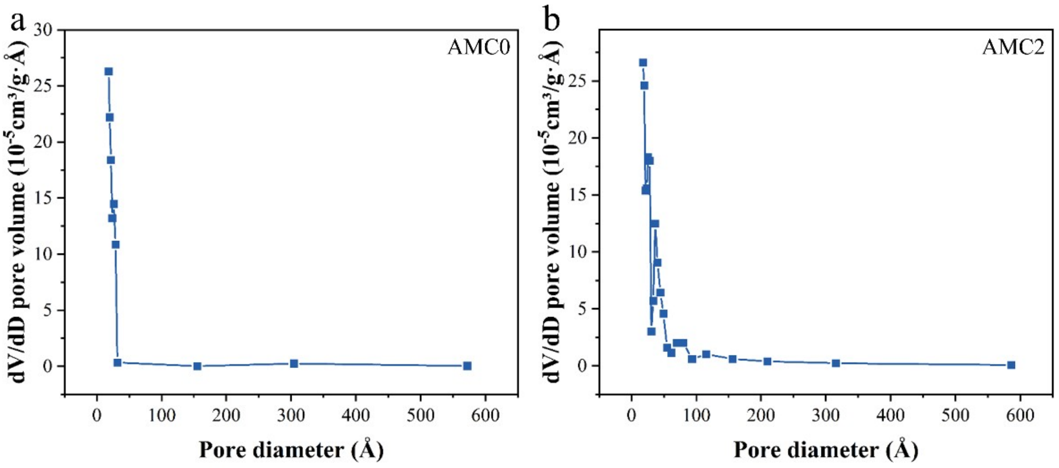

Figure S4. BJH pore-size distribution plots of (a) AMC0 and (b) AMC2 aerogel.

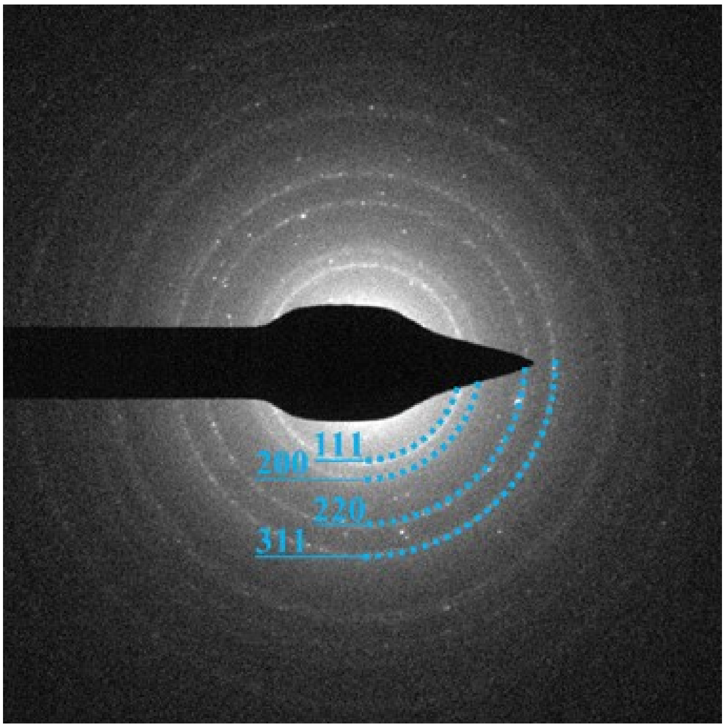

Figure S5. Selected area electron diffraction pattern (SAED) of the AMC aerogel.

Note: SAED characterization was newly supplemented for the AMC aerogel and has been added as Figure S5 in the Supporting Information, with its corresponding crystal planes accurately calibrated. Clear diffraction rings can be well indexed to the (111), (200), (220) and (311) lattice planes of face-centered cubic Au, verifying the successful decoration and high crystallinity of Au nanoparticles.[5] These findings are in excellent accordance with the XRD results in the manuscript. In sharp contrast, MXene exhibits only faint scattered diffraction spots rather than continuous diffraction rings. Such a phenomenon implies the loss of long-range structural periodicity, which aligns well with the XRD observation that the characteristic (002) peak of pristine MXene disappears in the aerogel. This structural feature stems from the exfoliation and random dispersion of MXene nanosheets within the CMC-Na network during aerogel fabrication, which effectively destroys the intrinsic long-range layered order of pristine MXene. Overall, the SAED results further corroborate the XRD analysis, collectively revealing the unique structural characteristics of the composite aerogel.

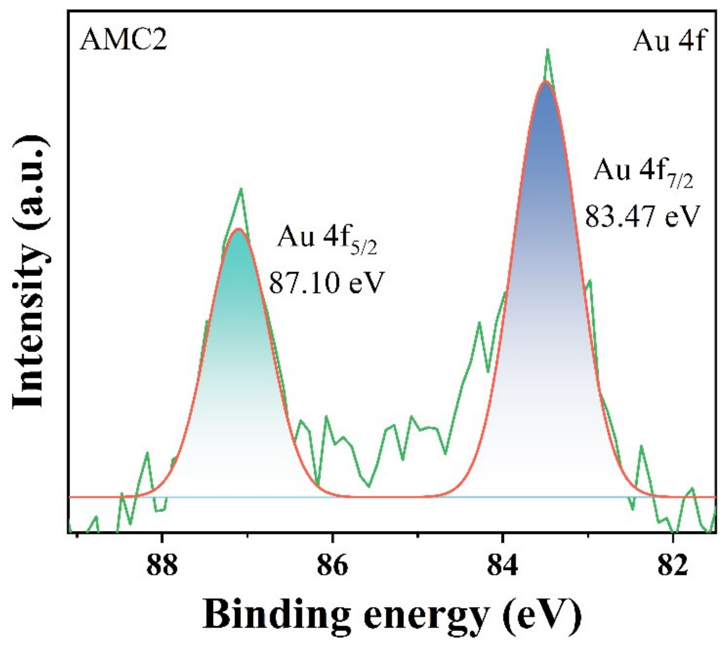

Figure S6. High-resolution Au 4f XPS spectra of AMC2 aerogel.

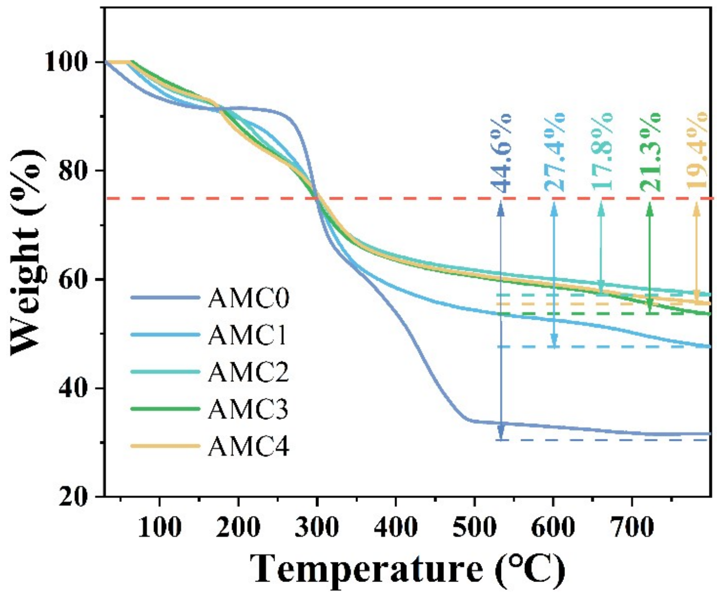

Figure S7. The TG curves of AMC0-AMC4.

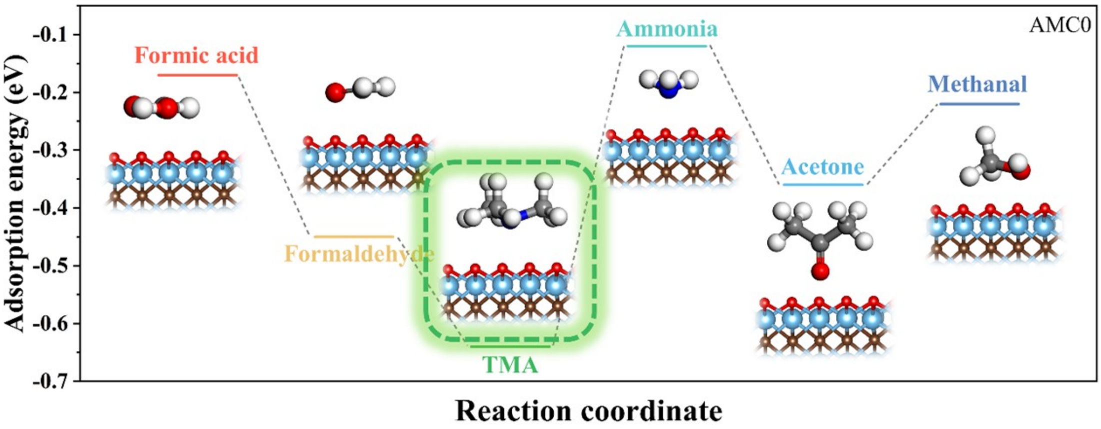

Figure S8. Adsorption energies of the AMC0 sensor for different gases.

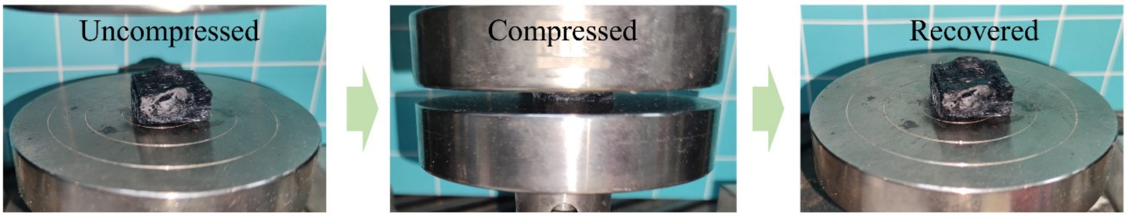

Figure S9. Images of the AMC aerogel before and after compression.

Note: A static uniaxial compression test was performed on the AMC aerogel, which was compressed to 50% of its original height at a compression rate of 10 mm·min^{-1} followed by the cessation of loading. Upon unloading, the aerogel achieved a complete structural rebound and recovered to its initial original height, exhibiting excellent mechanical compression recovery performance.

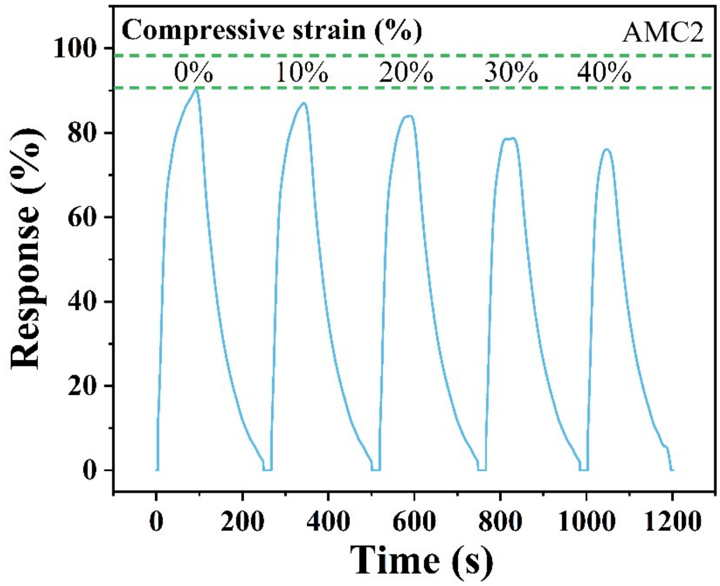

Figure S10. Response curves of aerogel under different compression levels to 100 ppm TMA.

Note: The response–recovery curves of the AMC aerogel were recorded under compressive strains from 0 to 40%. Even when subjected to a compressive strain of 40%, the aerogel still retains 83% of the response value compared with the uncompressed aerogel, demonstrating stable sensing performance under mechanical compression.

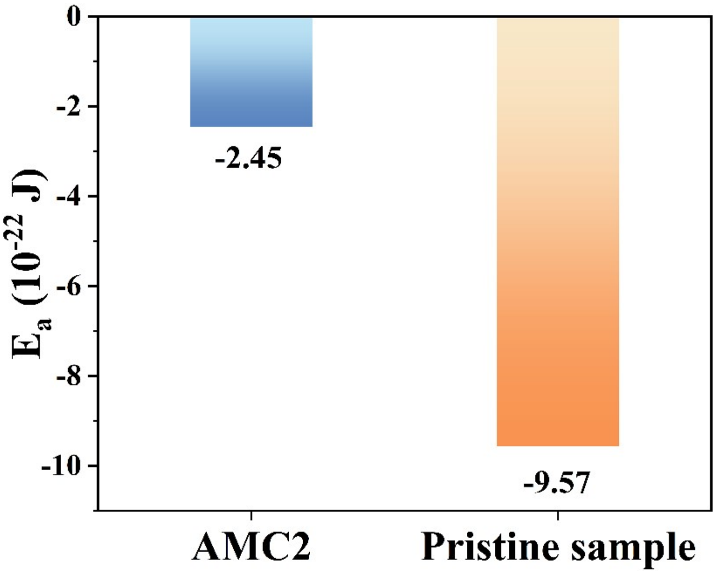

Figure S11. Activation energy (E_a) of the AMC2 aerogel and the pristine sample (AMC0).

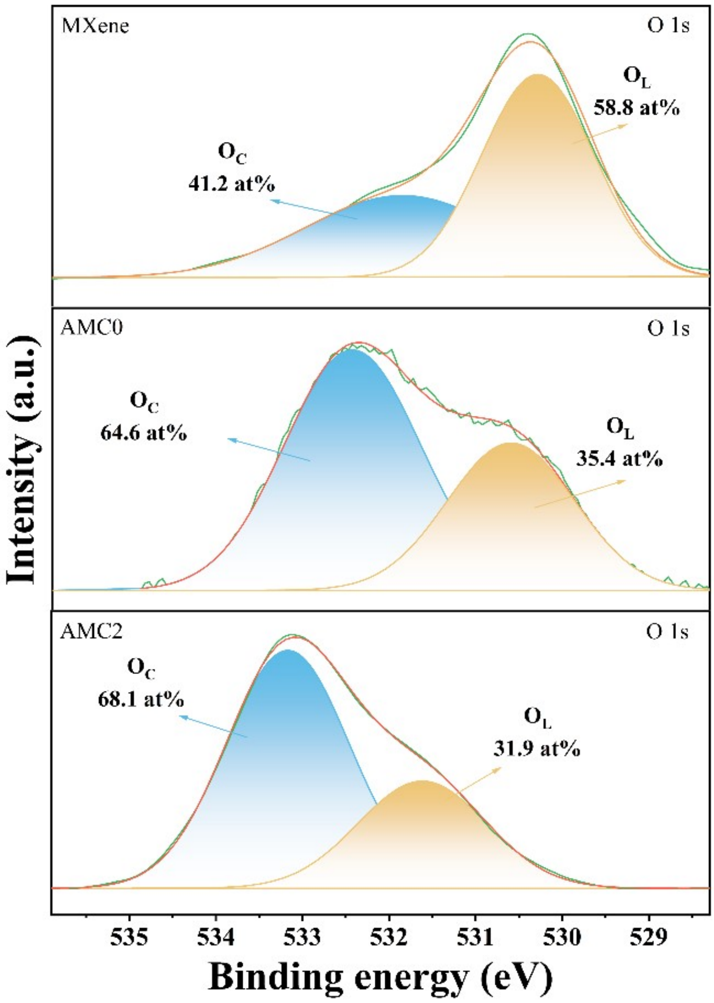

Figure S12. High-resolution O 1s XPS spectra of MXene, AMC0, and AMC2 aerogels.

Table S1. Specific surface areas of AMC aerogels.

Material	AMC0	AMC1	AMC2	AMC3	AMC4
BET surface area ($m^2 \cdot g^{-1}$)	9.01	12.85	19.91	1.23	1.00

Table S2. The comparison of TMA sensing performance between the AMC aerogels and previously reported sensors.

Sensing materials	Detection limit (ppm)	Response ($\lvert R_a - R_g \rvert / R_a$)/ Concentration (ppm)	Response/ Recovery time (s)	Selectivity	Operating temperature (°C)	Ref.
Rh/ZnO	1	11.3/10	63/106	1.22	180	[6]
AuCu@ $CuFe_2O_4$	1	42.8%/20	8/14	1.72	150	[7]
$Ti_3C_2T_x$ aerogel	5	26.2%/100	387/682	1.73	RT	Pristine sample
Au/ZnO	0.5	50/10	12/127	2	250	[8]
Au@Pt-α-Fe_2O_3	1	32/100	5/74	2.32	150	[9]
Au/WO_3	0.5	217.72/25	8/6	2.63	300	[10]
This work	5	90.7%/100	40.2/162.8	3.88	RT	This work

Reference

1. L. Jin, K. Yang, L. Chen, R. Yan, L. He, M. Ye, H. Qiao, X. Chu, H. Gao and K. Zhang, *Anal. Chem.*, 2023, **95**, 8859-8868.
2. E. Macke, I. Timrov, N. Marzari and L. C. Ciacchi, *J. Chem. Theory Comput.*, 2024, **20**, 4824-4843.
3. G. Kresse and J. Furthmüller, *Physical Review B*, 1996, **54**, 11169-11186.
4. A. Kumar, M. Kumar, R. Kumar, R. Singh, B. Prasad and D. Kumar, *Mater. Sci. Semicond. Process.*, 2019, **90**, 236-244.
5. J. Depciuch, M. Stec, M. Kandler, J. Baran and M. Parlinska-Wojtan, *Photodiagn. Photodyn. Ther.*, 2020, **30**, 101670.
6. Z. Li, C. Lou, G. Lei, G. Lu, H. Pan, X. Liu and J. Zhang, *Sens. Actuators, B*, 2022, **355**, 131347.
7. Y. Sun, D. Zhang, M. Tang, W. Liu, Y. Liu, J. Wang, G. Xi, H. Xiong and L. Zhang, *J. Alloys Compd.*, 2025, **1010**, 177662.
8. Y. Chen, Y. Li, B. Feng, Y. Wu, Y. Zhu and J. Wei, *Sens. Actuators, B*, 2022, **360**, 131662.
9. J. Shen, S. Xu, C. Zhao, X. Qiao, H. Liu, Y. Zhao, J. Wei and Y. Zhu, *ACS Appl. Mater. Interfaces*, 2021, **13**, 57597-57608.
10. C. Zhao, J. Shen, S. Xu, J. Wei, H. Liu, S. Xie, Y. Pan, Y. Zhao and Y. Zhu, *Food Chem.*, 2022, **392**, 133318.
